# Additional contributions to taxonomy, nomenclature and biogeography of the Turkish *Crataegus* (Rosaceae) taxa

**DOI:** 10.3897/phytokeys.122.33002

**Published:** 2019-05-16

**Authors:** Ali A. Dönmez, Sevgin Özderin

**Affiliations:** 1 Faculty of Science, Department of Biology, Section of Botany, Hacettepe University, Beytepe, Ankara 06532, Turkey Hacettepe University Ankara Turkey; 2 Muğla Sıtkı Koçman University, Köyceğiz Vocational School, Muğla 48000, Turkey Muğla Sıtkı Koçman University Muğla Turkey

**Keywords:** *
Crataegus
*, endemism, new variety, Rosaceae, Turkey

## Abstract

*Crataegusazarolus* L. has a wide distribution pattern from the western Mediterranean coasts to the eastern parts of Iran with several varieties adapted to local climatic conditions. Crataegusazarolusvar.senobaaensis**var. nov.** is described as a new variety from southeast Turkey with characteristic deep leaf sinuses, mostly 3–4 pairs of lobes and leaves ovate-oblong in outline. Two varieties of the species are accepted under the name of *Crataegusazarolus* and the correct names are published here. CrataegusmonogynaJacq.var.odemisii**var. nov.** is described from İzmir, in the western part of Turkey. This new variety is distinguished by its orange fruit colour. An outstanding disjunct distribution pattern has been discovered for the recently described species, *Crataegusyaltirikii* Dönmez. Updated descriptions and infraspecific identification keys for *Crataegusazarolus* and *Crataegusmonogyna* are given and pictures and distribution data for the new taxa are also supplied.

## Introduction

The genus *Crataegus* L. grows mostly in the northern hemisphere and prefers forest openings and open areas of steppe. The number of species in the genus is ca. 240 with around 170 species in the New World and 70 species in the Old World (Pojarkova 1939; Meikle 1966; Riedl 1969; Browicz 1972; Christensen 1992; Khatamsaz 1991; Gu and Spongberg 2003; Phipps et al. 2003; Dönmez 2013; Phipps 2018). Phylogenetic studies, based on DNA sequences from both chloroplast and nuclear markers by Campbell et al. (2007), Lo et al. (2007), Potter et al. (2007) and Li et al. (2012), clarified the intergeneric relationships amongst the genera of the tribe Maleae. Subsequent to the publication of a revision of the Old World *Crataegus* taxa by Christensen (1992), extensive field work, both in Turkey and neighbouring countries by Dönmez (2008), showed that the section Crataegus is extensively diversified, especially in Turkey (Dönmez 2004, 2005, 2007, 2013; Dönmez and Oybak Dönmez 2005). Although exhaustive field work and collections in the region have been undertaken, it is still possible to detect new populations, probably representing new taxa (e.g. Dönmez 2005; Shahbaz and Sadeq 2006; Dönmez 2007, 2013; Sharifnia et al. 2011).

**Figure 1.**
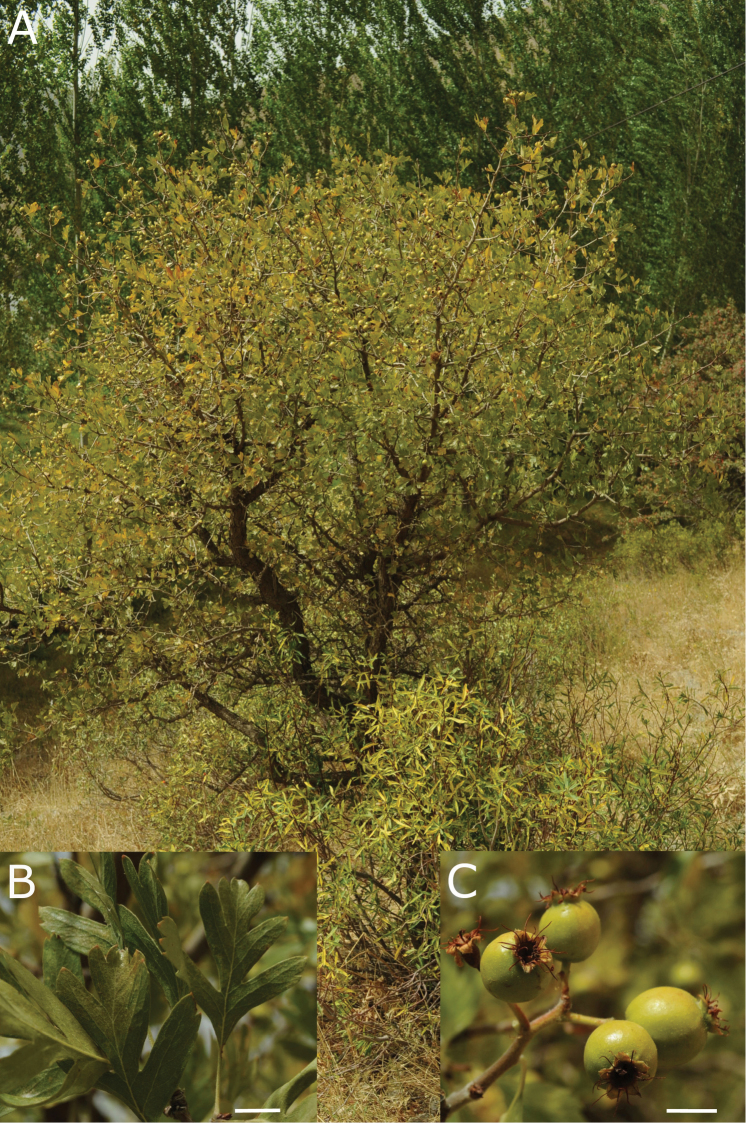
Crataegusazarolusvar.senobaaensis Dönmez. **A** View of fruiting bush in steppe habitat **B** leaves of short shoot **C** mature fruits. (*A.A. Dönmez* 18745). Scale bar: 1cm.

**Figure 2.**
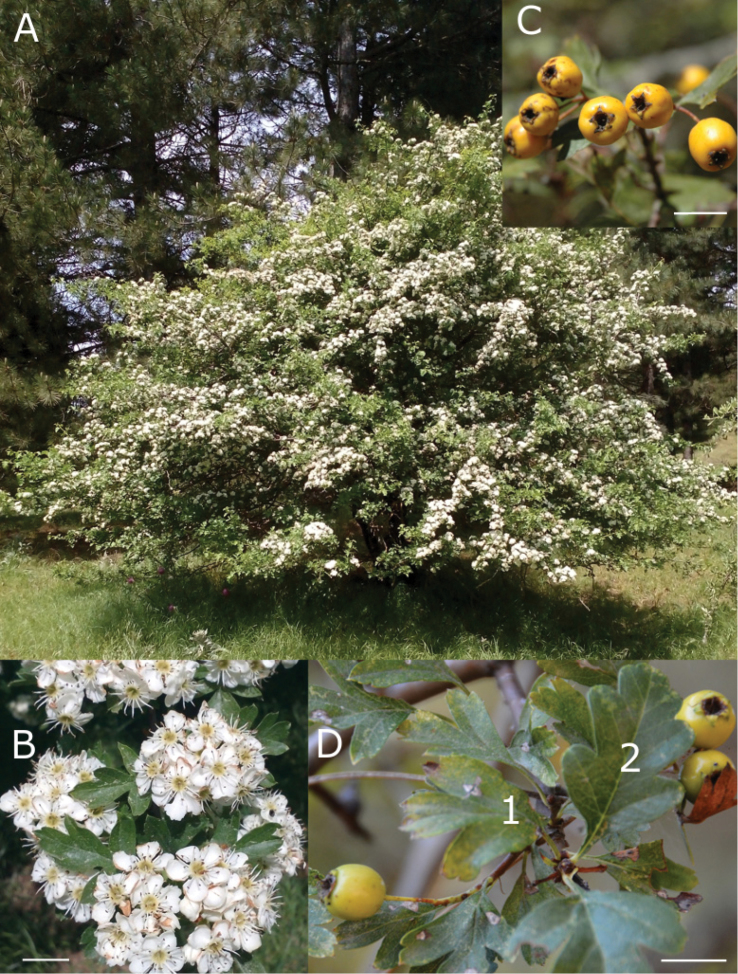
Crataegusmonogynavar.odemisii Dönmez & Özderin var. nov. **A** View of flowering individual in habitat **B** flowers **C** mature fruit **D1** leaf of fertile shoot **D2** leaf of short shoot. (**A–B***S.Özderin s.n.***C–D***A.A.Dönmez* 20263-*S.Özderin*). Scale bar: 1cm.

Ongoing studies both in the field and herbarium resulted in the discovery of a new variety (Figure 1) and an outstanding distribution pattern of a recently described species *C.yaltirikii* Dönmez. Moreover, during plant collection for the study on the photochemistry of the Aegean *Crataegus* species (Özderin et al. 2016), an unusual population of *C.monogyna* Jacq. with distinct pure yellow fruits has been discovered by the second author (Figure 2). Further, studies on the populations in the following years by the authors revealed that the fruit character is related to genetic factors and not temporary environmental conditions. Consequently, both specimens are described here as new varieties.

*Crataegusazarolus* L. is an economically important fruit plant and its fruits have been used for food, jam and other traditional cuisines in the area. Although there is an extensive distribution from Spain to Iran, this species does extensively diversify in Turkey. Two varieties have been described by Browicz (1972) and they have been reduced to synonym by Christensen (1992). Based on the observations of the taxa in the field, it was found that both of them should be accepted as distinct taxa.

## Materials and methods

The descriptions of CrataegusazarolusL.var.senobaaensis Dönmez and Crataegusmonogynavar.odemisii Dönmez & Özderin are based on field collections of the new taxa at Şırnak, Bitlis and İzmir, observation on the habitat and examination of ca. 600 herbarium specimens from E, EGE, G, HUB, ISTO, K, LE and W (acronyms follow Index Herbariorum; http://sweetgum.nybg.org/science/ih/) by the authors. The preliminary conservation status of the new taxa was assessed using the IUCN (2017) criteria, according to field observations in the type localities and their environs.

## Results

The descriptions of the species have been updated according to the relevant literature, field observations and collections, both from the mentioned herbaria and our own collections. The measurements are based on the herbarium materials.

### Taxonomic treatment

#### Crataegus azarolus

Taxon classificationPlantaeRosalesRosaceae

L., Sp. pl. 477. 1753.

##### Description.

Shrub or tree up to ca. 4 (-10) m tall. Twigs more or less lanate or lanate-tomentose. Thorns up to ca. 8 cm long, more or less stout. Buds 2–3 (-4.2) mm long, 2–3 (-4.8) mm in diameter. Leaf blades more or less coriaceous, more or less lustrous dark green and appressed-pubescent above, pale or greyish-green and glabrous or appressed pubescent beneath, attenuate, cuneate or rounded at base, lobes obtuse, acute or cuspidate, margin entire or serrate with more or less coarse teeth; basal pair of veins divergent, straight or convergent. Subterminal leaf blades of flowering shoots (10-) 15–30 (-80) × (7-) 10–25 (-70) mm, lobes 1–2 (-4) pairs, rarely absent, basal lobes sometimes extending to midrib, each lobe entire or with (1-) 2–3 (-6) teeth in distal half, lobe length 0.5–1 (-3) times to width; petiole (2-) 4–6 (-17) mm; stipules rarely absent or 3–5 (-10) × 0.5–1 (-3) mm, entire or with 1–3 teeth. Subterminal leaf blades of short shoots (10-) 15–30 (-70) × (10-) 15–25 (-50) mm, lobes 1–2 (-4) pairs, basal pair extending to midrib, basal lobe entire or with 3 (-6) teeth in the upper half; petiole (2-) 4–8 (-28) mm long; stipules mostly undeveloped or 2–3 (-5) × 0.5–1 mm. Leaf blades of elongate shoots (15-) 20–35 (-80) × 15–25 (-70) mm, lobes 1–3 (-4) pairs, basal pair entire or 1–4 (-8) teeth at upper half; petiole 2–10 (-20) mm; stipules 4–10 (-25) × (0-) 3–5 mm, with 3–5 (-10) teeth. Inflorescence (10-) 15–20 (-45) × 15–20 (-60) mm long, corymbose, (5-) 10–20 (-25) flowered, more or less lanate or lanate-tomentose; pedicels 2–5 (-10) mm; bracts 1–4 × 0.2–0.9 mm, caducous, linear or lanceolate, margin entire or denticulate with 1–6 teeth. Flowers (5-) 10–15 mm in diameter. Hypanthium 3–6 × 3–6 mm; sepals 1.2–3.5 × 1.6–3.9 mm, usually broadly triangular, margin entire, apex more or less acute; petals 3–7 × 4–7 mm; stamens 15–20 (-22), anthers purple; styles (1-) 2–3 (4). Fruit (6-) 8–12 (-35) mm, depressed-globose, globose or slightly pyriform, yellowish-green or orange, often tinged with red, when dried, often becoming dark red; the immature fruit crowned by the persistent erect or spreading sepals, at maturity sepals re-curved; pyrenes (4-) 5–7 × 6–8 (-20) mm, dorsally sulcate, ventrally smooth, hypostyle pilose.

#### Crataegus azarolus L. var. dentata

Taxon classificationPlantaeRosalesRosaceae

(Browicz) Dönmez comb. nov.

urn:lsid:ipni.org:names:77197215-1

##### Basionym.

CrataegusaroniaL.var.dentata Browicz, Notes Roy. Bot. Gard. Edinburgh 31: 324. 1972. TYPE [Turkey] Muğla: Marmaris, Bayır, 15 iv 1965, *P.H.Davis* 41136 (holotype: E!; isotype: K!).

#### Crataegus azarolus L. var. minuta

Taxon classificationPlantaeRosalesRosaceae

(Browicz) Dönmez comb. nov.

urn:lsid:ipni.org:names:77197216-1

##### Basionym.

Crataegusaroniavar.minuta Browicz, Notes Roy. Bot. Gard. Edinburgh 31: 324. 1972. TYPE: [Turkey] Hatay: 8 km from Belen towards Antakya, ca. 600 m elev., 6 v 1965, *Coode & Jones* 521 (holotype: E!).

#### Crataegus azarolus L. var. senobaaensis

Taxon classificationPlantaeRosalesRosaceae

Dönmez var. nov.

urn:lsid:ipni.org:names:77197217-1

Figure 1

##### Diagnosis.

This new variety differs from the other varieties (CrataegusazarolusL.var.minuta Browicz and CrataegusazarolusL.var.dentata Browicz and CrataegusazarolusL.var.aronia L.) by its deeply divided leaf sinuses, mostly 3–4 pairs of leaf lobes and mostly ovate-oblong leaves at outline.

##### Type.

TURKEY. Şırnak: 20 km from Uludere to Şırnak, above Şenoba, steppe, ca. 1250 m elev., 28. September 2002, *A.A.Dönmez* 11139, (holotype: HUB, isoptypes, HUB, EGE). Paratypes: Bitlis: Tatvan, Koruklu village, Yenitoprak district, 1734 m elev., hedge, 02.10.2013. *A.A.Dönmez* 18745-*K.Özgişi*. (HUB!).

##### Distribution.

**Figure 3.**
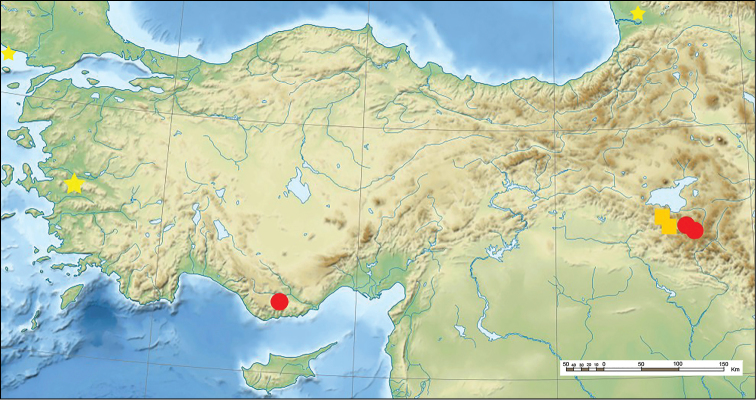
Distribution of the taxa. (square)Crataegusazarolusvar.senobaaensis; (star) Crataegusmonogynavar.odemisii var. nov. (circle) *Crataegusyaltirikii* (Near East topographic map-blank.svg).

(Figure 3). Crataegusazarolusvar.senobaaensis is endemic to southeast Turkey and it is known from two different locations.

##### Ecology and habitat.

Crataegusazarolusvar.senobaaensis grows in dry steppes from 1250 to 1735 m elevation.

##### Etymology.

The epithet denotes the type locality Şenoba.

##### Preliminary conservation status.

Crataegusazarolusvar.senobaaensis should be labelled as “Critically Endangered”, (CR B1+D) according to the IUCN (2017) threat categories. The area of occupancy is estimated to be smaller than 100 km^2^ and the number of examined mature individuals is less than 50. Besides this, all of the examined specimens are known from habitats which are not under threat.

### Infraspecific key Turkish taxa of *C.azarolus*

**Table:** 

1	Inflorescence and leaves up to 15 mm in length	**C.azarolusvar.minuta comb. nov.**
–	Inflorescence and leaves longer than 15 mm	**2**
2	Leaves one or rarely two shallowly lobed at apex; ovate or elliptic	**C.azarolusvar.dentata comb. nov.**
–	Leaves 2–4 lobed, lobes more than half of the lamina	**3**
3	Leaf lobes 1–2 times wide, sinuses 1/2 to 1/3 of lamina	** C. azarolus var. aronia **
–	Leaf lobes 3–4 times wide, leaves deeply pinnatisect	**C.azarolusvar.senobaaensis, var. nov.**

#### Crataegus yaltirikii

Taxon classificationPlantaeRosalesRosaceae

Dönmez Bot. J. Linn. Soc. 155(2): 239. 2007.

##### Lectotype.

[Turkey] Şırnak: Beytüşşebap, above Günyüzü village, opening of deciduous forest, 37°27'N, 043°09'E, 1495 m elev., 28.ix.2002; *A.A.Dönmez* 11143-*B.Mutlu* (designated by Dönmez 2007).

##### Distribution.

(Figure 3). *Crataegusyaltirikii* is a species native to Turkey where it is known from two locations namely Şırnak and Mersin which are far from each other, nearly 400 km distant.

##### Ecology and habitat.

*Crataegusyaltirikii* grows in *Quercusbrantii* Lindley forest openings at the type locality and amongst the maquis vegetation in Mersin, a recently discovered location.

##### Conservation status.

“LC” threat category was assessed for *Crataegusyaltirikii* in the description of the species (2005). Based on the new distribution pattern of the species, the threat category of the species was re-evaluated and “LC” is still the appropriate category for the species.

#### Crataegus monogyna

Taxon classificationPlantaeRosalesRosaceae

Jacq. Fl. Austriac. (Jacquin) 3: 50, t. 292, f. 1. 1775

##### Description.

Trees or shrubs up to 10 m. Twigs glabrous rarely villose. Thorny or thornless, thorns up to 25 (-70) mm. Buds 1.1–2.8 × 1.1–2.5 mm. Leaves ±coriaceous, ±greyish below, glabrous to villose or villose on veins beneath, attenuate to widely cuneate at base, lobes obtuse to acute, entire to incised serrate at margin. Subterminal leaf blades of flowering shoots 10–35 (-57) × 8–30 (-60) mm, lobes (0-) 1–2 (-3) pairs, basal sinuses close to midvein, angles of basal vein at or wider than 45°, rarely narrower, basal lobes entire or with 2–4 (-9) serrate teeth in distal 1/2 to 3/4, petiole (1-) 5–15 (-30) mm; stipules (1-) 3–10 (-16) × 0.2–0.4 mm, entire or irregularly glandular serrate with 1–5 (-8) teeth. Subterminal leaf blades of short shoots (10-) 15–35 (-57) × (8-) 15–30 (-55) mm, lobes (0-) 1–2 (-3) pairs, basal sinuses reach to half lamina or close to midvein, lobes entire or with (2-) 4–8 (-14) teeth in the distal 1/3 to 1/8, petiole 5–15 (-45) mm, stipules undeveloped or (1-) 2–3 mm, entire or with 2–4 teeth. Middle leaf blades of elongate shoots 20–40 (-60) × 20–40 (-65) mm, 2–3 (-4) pairs, basal lobes with (0-) 3–5 (-16) teeth in distal 1/4 to 1/8, petiole (6-) 10–20 (-25) mm; stipules (4-) 8–15 (-20) × 2–4 (-8) mm, entire or irregularly serrate with 2–10 (-35) teeth. Inflorescence (10-) 15–35 (-50) × 15–45 mm, corymbose, lax, (5-) 8–15 (-20) flowered, glabrous to villose, pedicels 4–10 (-32) mm, bracts 0.5–8 (-10) × 0.1–0.5 mm, deciduous, linear-lanceolate, entire or denticulate with 1–4 teeth. Flowers (5-) 8–12 mm in diameter; hypanthium 2–4 × 2–3 mm; sepals 1.2–4 × 1.2–2.6 mm, widely triangular margin entire, acute or obtuse; petals (3-) 4–5 (-7) × 4–6 (-7) mm; stamens (15-) 18–20, anthers maroon; styles 1. Fruit 5–11 × 4–7 (-10) mm, globose to cylindrical, red to dark red, rarely orange, glabrous or sparsely villose, flesh yellowish, juicy to mealy, sepals recurved at maturity; pyrenes 4–6 (-9) × 3–5 (-8) mm, 1 (-2), dorsally and ventro-laterally entire or striate, hypostyle glabrous.

#### Crataegus monogyna Jacq. var. odemisii

Taxon classificationPlantaeRosalesRosaceae

Dönmez & Özderin var. nov.

urn:lsid:ipni.org:names:77197218-1

Figure 2

##### Diagnosis.

This new variety differs from the other varieties (CrataegusmonogynaJacq.var.monogyna and Crataegusmonogynavar.lasiocarpa (Lange) K.I.Christ.) sensu Christensen (1992) by its orange fruit colour in contrast to red fruits in the former.

##### Type.

TURKEY. İzmir: Ödemiş, around Gölcük region, towards peak of Bozdağ, 1050–1120 m elev., 9 September, 2014, *A.A.Dönmez* 20263-*S. Özderin*, (holotype: HUB); isotypes: HUB, EGE). Paratypes: İzmir: Ödemiş, around Gölcük region, towards to peak of Bozdağ, 1050–1120 m elev., 8 October, 2013, *S. Özderin s.n.*; 22 May, 2015, *S. Özderin s.n.* (HUB).

##### Phenology.

flowering in May and fruiting in September-October.

##### Habitat.

in openings of *Pinusnigra* forest.

##### Distribution.

(Figure 3). Crataegusmonogynavar.odemisii is endemic to İzmir; it has a narrow distribution at Gölcük.

##### Ecology and habitat.

Crataegusmonogynavar.odemisii grows in openings of *Pinusnigra* forest between 1050 and 1120 m elevation. The basic vegetation type is maquis at lower elevations of the area. Besides this, *Pinusnigra* forest replaces it at higher altitudes and steppe vegetation dominates above the tree zone.

##### Etymology.

The epithet of this new variety denotes the collection area, Ödemiş.

##### Preliminary conservation status.

Crataegusmonogynavar.odemisii should be assigned to “Critically Endangered”, (CR B2ab(i,ii,iii,iv), D) according to the IUCN (2017) threat categories. The area of occupancy is estimated to be less than 10 km^2^ and the examined specimens are known only from alongside the road. The location is close to the picnic area of the Gölcük Lake and is under threat from fire, cutting and other anthropogenic effects.

##### Discussion.

Two new varieties have been published by Browicz (1972) from Turkey under the species name of *C.aronia* L. These two varieties were reduced to synonym by Christensen (1992) with their published name and he accepted the name *Crataegusazarolus* instead of *C.aronia*. Taxonomic decisions of Christensen have been based solely on herbarium material. During the taxonomic revision of the genus *Crataegus*, the first author had the opportunity to observe these varieties in their habitats alongside the complete set of morphological variations of the species. Moreover, morphological studies on the large set of herbarium material in the above-mentioned herbaria and fieldwork from Greece to Iran provided more opportunity to observe all kinds of variation of *C.azarolus* and the closely related taxa. Consequently, based on field observations and herbarium studies on the collected materials, these two varieties, namely CrataegusazarolusL.var.dentata (Browicz) Dönmez and CrataegusazarolusL.var.minuta (Browicz) Dönmez should be accepted as distinct taxa and they should be given as new combinations under the species name of *Crataegusazarolus*.

*Crataegusmonogyna* is one of the most polymorphic species amongst the Eurasian *Crataegus* taxa with respect to leaf morphology and indumentum. Due to local variations of the species and species concepts by the authors who studied *Crataegus*, many new taxa have been described. In addition, new combinations and alteration of their status have been made. Based on these taxonomic and nomenclatural novelties, both taxonomic and nomenclatural synonyms of about 200 names have been listed by Christensen (1992). In *C.monogyna*, extensive variations in leaf morphology and indumentum are present, whereas variations in fruit colour are limited. Fruit colour of *C.monogyna* is clearly red and/or with degrees of red. Specimens of the new variety, C.monogynavar.odemisii are yellow. Red and various degrees of red colour for fruit of infraspecific *C.monogyna* taxa have been observed by the second author and they have hundreds of representative specimens for these fruit colours in the above-mentioned herbaria. According to observations on the fruit colour of single pyrened *Crataegus* taxon, it is a constant character and unique in the infraspecific taxa of *C.monogyna*. Hence, it is worthwhile accepting this population as a separate taxonomic status, as a variety.

*Crataegusyaltirikii* Dönmez is a recently described new species from southeast Turkey and we found a new population of the species on the Taurus Mountain ranges, nearly 400 km away from the type locality. Climatic conditions of these two localities are different; the type locality is a cold and snowy area, whereas the recently discovered locality is characterised by hot and dry summers, rainy and warm winters. We have not yet obtained molecular works on these disjunct populations. Besides this, we assume that these populations should be local ecotypes of the species.

### Infraspecific key to Turkish taxa of *Crataegusmonogyna*

**Table:** 

1	Fruit orange yellow; fertile leaf lobes with only few teeth, short shoot leaf lobes with many teeth	**C.monogynavar.odemisii var. nov.**
–	Fruit red to dark red; teeth of fertile and short shoot leaf lobes are similar	**2**
2	Leaves, twigs and fruits sparsely villous or glabrous	*** C. monogyna ***
–	Leaves, twigs and fruits densely villous	*** C. lasiocarpa ***

### Selected additional specimens examined

**CrataegusazarolusL.var.dentata (Browicz) Dönmez**

TURKEY. Muğla: Datça, Taşlıca village, Karayurt district, limestone, 36°37.7’N, 28°6.1’E 208 m elev., 1 December 2001, *A.A.Dönmez* 10412 -*S.Işık* (HUB!). İçel: Tarsus, Kadıncık I Dam, 500 m elev., 11 May 1990, *Y.Gemici* 5480 (EGE!); Tarsus, Beylice village, 600 m elev., 10 August 1990, *A. Güner* 7951-*H.Karaca* (HUB!).

**CrataegusazarolusL.var.minuta (Browicz) Dönmez**

TURKEY. Denizli: around Pamukkale, 9 May 1975, *Browicz & Zielinskii* 104 (E!, LE!). Antalya: Kaş, Lengumen village, around Belpınarı, 1100 m elev., limestone, 9 September 1992, *A.A.Dönmez* 2963 (HUB!); Elmalı, y. 1300 m elev., 4 June 1961, *Howard C.Stutz* 1512 (W!). Konya: Ermenek, E of Ermenek ca. 1200 m elev., 27 May 1978, *M.Vural* 700 (ANK!). Mersin: Mut, Magras Mountain, 1100 m elev., 750 m elev., 11 May 1965, *Coode & Jones* 750 (E!, ISTO!). Kahramanmaraş: from Kahramanmaraş to Zeytin, Ahır Mountain, 1100 m elev., 8 May 1934, *Balls* 991 (E!). Kilis: 2 km from Kilis to Radar, slopes, *Quercus* çalılığı, ca. 800 m elev., 2 June 2000, *A.A.Dönmez* 7812 (HUB!).

**Crataegus monogyna Jacq. var. monogyna**

TURKEY. Edirne: Enez, Kılıçbey village, *Quercus* forest, 40°46.3"N, 26°32.61"E, 30 m elev., 8 May 2001, *A.A.Dönmez* 8704 (HUB!). Kırklareli: Demirköy Değirmendere district, 41°49.61"N, 27°45.25"E, 265 m elev., 9 May 2001, *A.A.Dönmez* 8778 (HUB!). Tekirdağ: Ganos Mt., Akçahalil village, ca. 500 m elev., 5 November 1999, *A.A.Dönmez* 6795 (HUB!). Çanakkale: Gelibolu, Bolayır, Koruköy, 40°33.65"N, 26°48.41"E, 60 m elev., 7 May 2001, *A.A.Dönmez* 8681 (HUB!). İstanbul: Büyükdere, Cumhuriyet street, amongst *Pinusnigra*, 41°9.6"N, 29°2.63"E, 50 m elev., 10 November 2001, *A.A.Dönmez* 10400 (HUB!). Kocaeli: Gebze, TÜBİTAK MAM campuse, ca. 100 m elev., 17 December 2002, *A.A.Dönmez* 11097. Bilecik: Taşçılar village, 40°14.5"N, 29°53.93"E, 600 m elev., 5 May 2001, *A.A.Dönmez* 8648 (HUB!). Bolu: Mengen, Çapak stream, *Pinusnigra* opening, ca. 550 m elev., 17 May 2002, *A.A. Dönmez* 10602 (HUB!). Ankara: Nallıhan, Kabaca village, 40°19.56"N, 31°21.19"E, 774 m elev., 24 August 2001, *A.A.Dönmez* 10004 (HUB!). Ankara: Ayaş road, amongst *Quercus*-*Pinus* plantation, 40°4.78"N, 32°27.95"E, 1050 m elev., 1 June 2001, *A.A.Dönmez* 8918 (HUB!). Karabük: between Karabük and Eskipazar, 41°19.05"N, 32°40.18"E, 289 m elev., 17 May 2002, *A.A.Dönmez* 10580 (HUB!). Kastamonu: Boyalı, Bahçeçiçek village, *Abiesnordmanniana* opening, 41°9.12"N, 33°18.85"E, 993 m elev., 5 June 2001, *A.A.Dönmez* 9201 (HUB!). Amasya: Suluova, 630 m elev., M&D Zohary 2165 (E!). Tokat: between Koyulhisar and Reşadiye, *Quercus*-*Pinus* opening, 40°22.25"N, 37°33.2"E, 505 m elev., 25 May 2002, *A.A.Dönmez* 10569 (HUB!). Artvin: west of the city, 500 m elev., 3 June 1993, *A.A.Dönmez* 3244 (HUB!). Çanakkale: Biga, Gerlengeç village, ca. 5 m elev., *Quercus*-*Fraxinus* scrub, 07 April 1999, *A.A.Dönmez* 7532 (HUB!). İzmir: Bozdağ, from Ödemiş to Bozdağ *Quercus* scrub, 38°17.16"N, 28°3.18"E, 990 m elev., 4 April 2001, *A.A.Dönmez* 8356 (HUB!). Aydın: Nazilli, Yağdere village, 37°55.43"N, 28°12.85"E, 312 m elev., 4 April 2001, *A.A.Dönmez* 8339 (HUB!). Balıkesir: Akbaş village, *Quercus*-*Juniperus* scrub, 39°40.08"N, 27°31.96"E, 390 m elev., 6 May 2001, *A.A.Dönmez* 8662 (HUB!). Bilecik: Söğüt, *Quercus* opening, 39°58.16"N, 30°7.15"E, 1100 m elev., 5 April 2001, *A.A.Dönmez* 8378 (HUB!). Manisa: Kula, Sandal village, 38°59.16"N, 28°34.05"E, 504 m elev., 21 August 2001, *A.A.Dönmez* 9931 (HUB!). Kütahya: from Harmancı to Tavşanlı, 39°35.45"N, 29°24.76"E, 872 m elev., 22 August 2001, *A.A.Dönmez* 9957 (HUB!). Uşak: between Delihıdırlı and Karahallı, 38°20.58"N, 29°33.85"E, 879 m elev., 20 August 2001, *A.A.Dönmez* 9927 (HUB!). Afyon: Çay, 1000 m elev., 9 August 1992, *A.A.Dönmez* 2905 (HUB!). Denizli: Acıgöl, rocky places, 37°49.4"N, 29°45.26"E, 845 m elev., 23 May 2001, *A.A.Dönmez* 8909 (HUB!). Isparta: Eğirdir, Akpınar village, 37°50.2"N, 30°51.18"E, 1100–1400 m elev., 1 April 2001, *A.A.Dönmez* 8287 (HUB!). Konya: Beyşehir, Yeşildağ village, 37°34.01"N, 31°32.21"E, 1210 m elev., 19 August 2001, *A.A.Dönmez* 9910 (HUB!). Ankara: Beytepe, ca. 950 m. elev. 24 September 2000, *A.A.Dönmez* 8092 (HUB!). Kırıkkale: Delice, Baraklı village, 17 August 1993, *A.A.Dönmez* 3927 (HUB!). Yozgat: Saray, 39°43.23"N, 34°42.2"E, 1100 m elev., 4 April 2001, *A.A.Dönmez* 8388 (HUB!). Adana: Himmetli, Saimbeyli, Davis 26652 (E!). Adıyaman: Gölbaşı, between Meydan and Hamzalar village, 37°52.81"N, 37°40.33"E, 1035 m elev., 15 September 2001, *A.A.Dönmez* 10126 (HUB!). Elazığ: from Elazığ to Malatya, Gülmahmut village, 38°33.78"N, 39°3.03"E, 1188 m elev., 21 April 2002, *A.A.Dönmez* 10518 (HUB!). Erzincan: Kemah, Eriç-Tuztaşı, ca. 800–900 m elev., 28 May 1998, *A.A.Dönmez* 6451 (HUB!). Malatya: Elazığ road, Kapıkaya village, 38°20.65"N, 38°33.3"E, 930 m elev., 21 April 2002, *A.A.Dönmez* 10515 (HUB!). Aydın: from Nazilli to Ödemiş, Hisarcık village, 38°055"N, 28°23.45"E, 380 m elev., 22 September 2001, *A.A.Dönmez* 10156 (HUB!). Denizli: Babadağ, Göçükoluk pasture, 37°48.16"N, 28°51.1"E, 1300 m elev., 23 May 2001, *A.A.Dönmez* 8898(HUB!). İzmir: Beydağ, Mutalar village, 38°4.4"N, 28°14.35"E, 260 m elev., 22 September 2001, *A.A.Dönmez* 10158 (HUB!). Muğla: from Muğla to Denizli, 37°11.09"N, 28°37.63"E, 805 m elev., 21 September 2001, *A.A.Dönmez* 10152. Antalya: Kaş, Lengumen village, 1100 m elev., 9 September 1992, *A.A.Dönmez* 2965 (HUB!). Konya: from Beyşehir to Akseki, 37°32.06"N, 31°34.45"E, 1180 m elev., 20 April 2001, *A.A.Dönmez* 8613 (HUB!). Mersin: Fındıkpınarı, Turunçlu, 36°49.75"N, 34°26.2"E, 570 m elev., 8 March 2001, *A.A.Dönmez* 8186-*B. Mutlu* (HUB!). Adana: Çamardı, Yelyutan village, 1300 m elev., 18 May 1993, *A.A.Dönmez* 3183 (HUB!). Kahramanmaraş: between Andırın and Gebez, 1300 m elev., 21 May 1993, *A.A.Dönmez* 3205 (HUB!). Osmaniye: Düziçi, Düldül Mt., Çitli village, 37°19.16"N, 36°29.55"E, 1012 m elev., 22 June 2004, *A.A.Dönmez* 12041 (HUB!). Hatay: Belen, 36°31.41"N, 36°15.26"E, 1300 m elev., 28 June 2001, *A.A.Dönmez* 9469 (HUB!). Mardin: from Mardin to Diyarbakır, 1000 m elev., Davis 28718 (E!). Şırnak: from Şırnak to Eruh, 37°39.28"N, 42°19.25"E, 1550 m elev., 28 September 2002, *A.A.Dönmez* 11138 (HUB!).

**Crataegusmonogynavar.lasiocarpa (Lange) K.I.Christ.**

TURKEY. Edirne: İpsala, Sarpdere, 40°53.23"N, 26°24.68"E, 60 m elev., 8 May 2001, *A.A.Dönmez* 8732 (HUB!). Tekirdağ: Hayrabolu, Çarıklı village, ca. 150 m elev., 19 June 1999, *A.A.Dönmez* 6941 (HUB!). Bolu: between Göynük and Mudurnu, 40°26.9"N, 30°54.08"E, 1105 m elev., 7 July 2003, *A.A.Dönmez* 11662(HUB!). Aydın: Karacasu, 37°44.01"N, 28°37.45"E, 422 m elev., 3 March 2001, *A.A.Dönmez* 8332 (HUB!). Balıkesir: Susurluk, 39°46.31"N, 28°2.56"E, 300 m elev., 6 May 2001, *A.A.Dönmez* 8658 (HUB!). Kütahya: Sabuncu, Fındık village, 39°33.33"N, 30°13.13"E, 962 m elev., 23 August 2001, *A.A.Dönmez* 9984 (HUB!). Ankara: Botanik Park, ca. 1000 m elev., 06 October 2000, *A.A.Dönmez* 8140. Tunceli: Pülümür, Gökçekonak village, 39°23.65"N, 39°50.13"E, 1252 m elev., 1 June 2002, *A.A.Dönmez* 10871 (HUB!). Muğla: Fethiye, Kemer, Kayacık village, 890 m elev., 8 September 1992, *A.A.Dönmez* 2961 (HUB!). Antalya: Kaş, 1130 m elev., limestone, 9 September 1992, *A.A.Dönmez* 2967 (HUB!). Adana: Kozan, 800 m elev., 18 May 1993, *A.A.Dönmez* 3191 (HUB!). Osmaniye: Düziçi, Düldül Mt., Çitli village, 37°19.16"N, 36°29.55"E, 1012 m elev., 22 June 2004, *A.A.Dönmez* 12045 (HUB!).

## Supplementary Material

XML Treatment for Crataegus azarolus

XML Treatment for Crataegus azarolus L. var. dentata

XML Treatment for Crataegus azarolus L. var. minuta

XML Treatment for Crataegus azarolus L. var. senobaaensis

XML Treatment for Crataegus yaltirikii

XML Treatment for Crataegus monogyna

XML Treatment for Crataegus monogyna Jacq. var. odemisii
